# Availability of healthy foods, fruit and vegetable consumption, and cognition among urban older adults

**DOI:** 10.1186/s12877-023-04003-z

**Published:** 2023-05-17

**Authors:** Jinshil Hyun, Mindy J. Katz, Carol A. Derby, Nelson Roque, Elizabeth Muñoz, Martin J. Sliwinski, Gina S. Lovasi, Richard B. Lipton

**Affiliations:** 1 The Saul R. Korey Department of Neurology, Albert Einstein College of Medicine, 1300 Morris Park Avenue, Bronx, NY 10461 USA; 2 Department of Psychology, University of Central Florida, Orlando, FL USA; 3 Department of Human Development and Family Sciences, University of Texas Austin, Austin, TX USA; 4 Department of Human Development and Family Studies, The Pennsylvania State University, University Park, PA USA; 5 Center for Healthy Aging, The Pennsylvania State University, University Park, PA USA; 6 Department of Epidemiology and Biostatistics, Drexel University, Philadelphia, PA USA; 7 Urban Health Collaborative, Dornsife School of Public Health, Drexel University, Philadelphia, PA USA; 8 Headache Center, Montefiore Medical Center, Bronx, NY USA

**Keywords:** Availability of healthy foods, Subjective and objective measures, Fruit and vegetable consumption, Ecological momentary assessments, Processing speed, Spatial working memory, Short-term memory binding

## Abstract

**Background:**

. Although prior studies have examined the associations between neighborhood characteristics and cognitive health, little is known about whether local food environments, which are critical for individuals’ daily living, are associated with late-life cognition. Further, little is known about how local environments may shape individuals’ health-related behaviors and impact cognitive health. The aim of this study is to examine whether objective and subjective measures of healthy food availability are associated with ambulatory cognitive performance and whether behavioral and cardiovascular factors mediate these associations among urban older adults.

**Methods:**

. The sample consisted of systematically recruited, community-dwelling older adults (N = 315, mean age = 77.5, range = 70–91) from the Einstein Aging Study. Objective availability of healthy foods was defined as density of healthy food stores. Subjective availability of healthy foods and fruit/vegetable consumption were assessed using self-reported questionnaires. Cognitive performance was assessed using smartphone-administered cognitive tasks that measured processing speed, short-term memory binding, and spatial working memory performance 6 times a day for 14 days.

**Results:**

. Results from multilevel models showed that subjective availability of healthy foods, but not objective food environments, was associated with better processing speed (estimate= -0.176, p = .003) and more accurate memory binding performance (estimate = 0.042, p = .012). Further, 14~16% of the effects of subjective availability of healthy foods on cognition were mediated through fruit and vegetable consumption.

**Conclusions:**

. Local food environments seem to be important for individuals’ dietary behavior and cognitive health. Specifically, subjective measures of food environments may better reflect individuals’ experiences regarding their local food environments not captured by objective measures. Future policy and intervention strategies will need to include both objective and subjective food environment measures in identifying impactful target for intervention and evaluating effectiveness of policy changes.

**Supplementary Information:**

The online version contains supplementary material available at 10.1186/s12877-023-04003-z.

About 6.5 million Americans over the age of 65 years are living with Alzheimer’s dementia today, with this number expected to grow to 12.7 million by 2050 [1]. Alzheimer’s Disease and related dementias (ADRD) result in substantial familial, societal, and economic burden. In contrast, preserving cognitive health into old age helps individuals maintain a high quality of life and autonomy. Prior studies have found that individuals’ behavioral and cardiovascular risk factors such as physical inactivity, low social contact, excessive alcohol consumption, smoking, diabetes, and hypertension are important modifiable risk factors for cognitive impairment and dementia [2, 3]. However, given that these factors are difficult to modify without supportive environments, there is growing interest in examining how neighborhood environments can help protect against cognitive impairment [4–7]. Among various neighborhood characteristics, local food environments are especially important because they may be associated with cognitive health either directly or through effects on dietary habits, routine activity patterns, or vascular risk factors. The overall goal of this study is to investigate whether and how availability of healthy foods is associated with cognition among older adults.

## Objective and subjective measures of local food environments

Previous studies have examined whether local food environments, assessed using either objective or subjective measures, were associated with various health outcomes and health-related behaviors [8–10]. Objective food environment measures typically use publicly available data to assess food environments (e.g., store density, access to supermarkets) within certain administrative boundaries (e.g., census tract) or geographical distances (e.g., buffer area of 250 ~ 1600 m) around individuals’ home locations. However, some limitations should be considered in using objective measures. First, there may be individual differences in what people consider as a ‘neighborhood’. Prior work suggested that there was large variability in the perception of neighborhood sizes across individuals [11]. Second, objective data cannot take into account factors (e.g., satisfaction with food availability or quality, transportation) [12] that may contribute to individuals’ actual experiences regarding how they navigate and interact with their local food environments. Therefore, subjective measures of local food environments may provide additional information not captured by objective measures and may better reflect individuals’ actual exposures and experiences with local food environments [13–15].

For late-life cognitive outcomes, only a few studies [16, 17] have examined the associations of objective or subjective availability of healthy foods with late-life cognition. FangFang and colleagues [16] found that objective measures of availability of healthy foods were associated with lower Mini Mental State Examination (MMSE) scores among females, but Finlay and colleagues did not find significant associations between objectively assessed healthy food environments and global cognition [18]. Tani and colleagues [17] found that both objective and subjective measures of availability of healthy foods were associated with decreased dementia incidence, with stronger effects of subjective rather than objective neighborhood measures [17]. However, the evidence on the differential roles of objective and subjective measures of food environments in late-life cognition is still lacking.

In addition, the socio-ecological model [19, 20] posits that dynamic societal and ecological contexts, which individuals are exposed to, play a role in driving health-related behavior and health consequences. There have been important advances in our understanding of individual factors that may lead to cognitive impairment and dementia, including fruit and vegetable consumption, physical activity, and cardiovascular factors [3, 21, 22]. However, little is known about how these individual factors are contextualized within the environment. As behavioral interventions to promote cognitive health would not be successful without supportive environments, it would be important to examine the role of local food environments in shaping health-related behaviors and cognitive health. The current study aimed to examine (1) whether both objective and subjective food environments were associated with late-life cognition, and (2) whether the associations between food environments and cognition were mediated by behavioral (i.e., fruit and vegetable consumption, walking) and cardiovascular risks (i.e., diabetes, hypertension).

## Cognitive domains related to early cognitive impairment

For public health perspectives, it is important to identify modifiable factors for subtle cognitive impairment that is associated with accelerated cognitive aging or clinical dementia. Identifying these modifiable factors may be hard using global or composite cognitive scores, because specific risk/protective factors may only be related to particular cognitive domains [6]. Indeed, some cognitive domains may be sensitive to earlier stages of cognitive aging and dementia. Processing speed is regarded as elementary cognitive operation that influences the efficiency of more complex cognitive abilities [23]. Some researchers suggest that processing speed may be a fundamental cause of age-related declines in other cognitive capabilities [24, 25]. Working memory is responsible for temporarily holding sensory information and manipulating it. There may be two components to the age-related decline in working memory performance: one emphasizing the storage of information and the other maintaining associations that bind individual features (e.g., color) to objects (e.g., shape) [26]. There is evidence that deficits in short-term memory binding performance may be a preclinical marker for Alzheimer’s disease [27].

Prior studies that examined the effect of the food environments on late-life cognition have used dementia status [17] or global cognitive measures (i.e., MMSE) [16] as outcomes. However, whether food environments affect specific cognitive domains remains to be established. In the current study, we aimed to examine the associations of availability of healthy foods with measures of processing speed and working memory performance, which are sensitive to age-related decline [25, 26] and/or age-related neuropathology (e.g., preclinical dementia) [27].

The Einstein Aging Study (EAS) provided the opportunity to examine associations between availability of healthy foods and late-life cognition in a diverse community-dwelling sample of urban older adults in Bronx, NY. In measuring cognitive performance, we employed ecological momentary assessment (EMA) methods using smartphones to conduct frequent and repeated cognitive testing in people’s everyday lives [28]. This approach has several advantages over the traditional, laboratory-based, single-shot cognitive assessments. By assessing cognitive performance in settings where people use their cognitive abilities, we increase ecological validity. In addition, repeated testing of cognition allows us to improve measurement precision and reliability of systematic between-person differences in cognitive performance by statistically modeling or canceling out the effects of random and systematic within-person variability. We hypothesized that greater availability of healthy foods would be associated with better cognitive performance assessed using EMA smartphone-administered cognitive tasks. We also hypothesized that the associations between availability of healthy foods and cognition would be mediated by behavioral (i.e., fruit and vegetable consumption, walking) and cardiovascular (i.e., hypertension, diabetes) factors.

## Methods

The EAS is a longitudinal population-based study of older adults in the Bronx, NY. Data for the present study were derived from individuals’ first annual EMA assessments completed between May 2017 and March 2020. A description of the study cohort and protocol can be found elsewhere [29, 30]; details relevant for the present study are provided below.

### Participants

Participants included 315 older adults with the mean age of 77.5 (range = 70 to 91). They were systematically recruited from registered voter lists in the Bronx, NY [29, 30]. Eligible participants were aged 70 and older, ambulatory, fluent in English, and residents of Bronx County, NY. Exclusion criteria included significant hearing or vision loss, current substance abuse, severe psychiatric symptoms that may interfere with testing, chronic medicinal use of opioids or glucocorticoids, treatment for cancer within the last 12 months, or a diagnosis of dementia at enrollment.

### Procedure

During recruitment, introductory letters were mailed to individuals from sampling frames generated from the voter lists. A research assistant followed up with a phone call to establish eligibility and schedule a clinic visit. At the baseline clinic visit, written consent was obtained and participants completed a conventional neuropsychological battery and questionnaires about demographics, medical history, family history, and other socio-behavioral factors. Then participants were given surveys assessing subjective neighborhood quality, dietary behaviors, physical activity, and other psychosocial characteristics to complete at home and return on their next visit. Participants returned to the clinic site to be trained on the use of study smartphone in which surveys for EMA were administered. Written informed consent for the EMA protocol was obtained in this initial clinic visit. Beginning the day after their clinic visit, participants began a 2-day run-in practice phase, followed by a 14-day EMA protocol. During the 14-day EMA protocol, participants completed a brief smartphone morning survey upon waking, beeped surveys at 4 quasi-random times during a day, and a bedtime survey at the end of each day for 14 days. The average time between beeped assessments was approximately 3.5 h. For each EMA assessment, participants completed a smartphone survey about their psychosocial states, immediately followed by several brief ambulatory cognitive tasks including Symbol Match, Color-Shape Binding, and Grid Memory tasks described in detail below. Participants who satisfactorily completed the entire study protocol could receive up to $160. The current study used data from the 14-day EMA protocol without the run-in data. The Institutional Review Board of the Albert Einstein College of Medicine approved the study protocol, and all participants provided informed consent.

### EMA mobile cognitive tasks

**Fig. 1:**
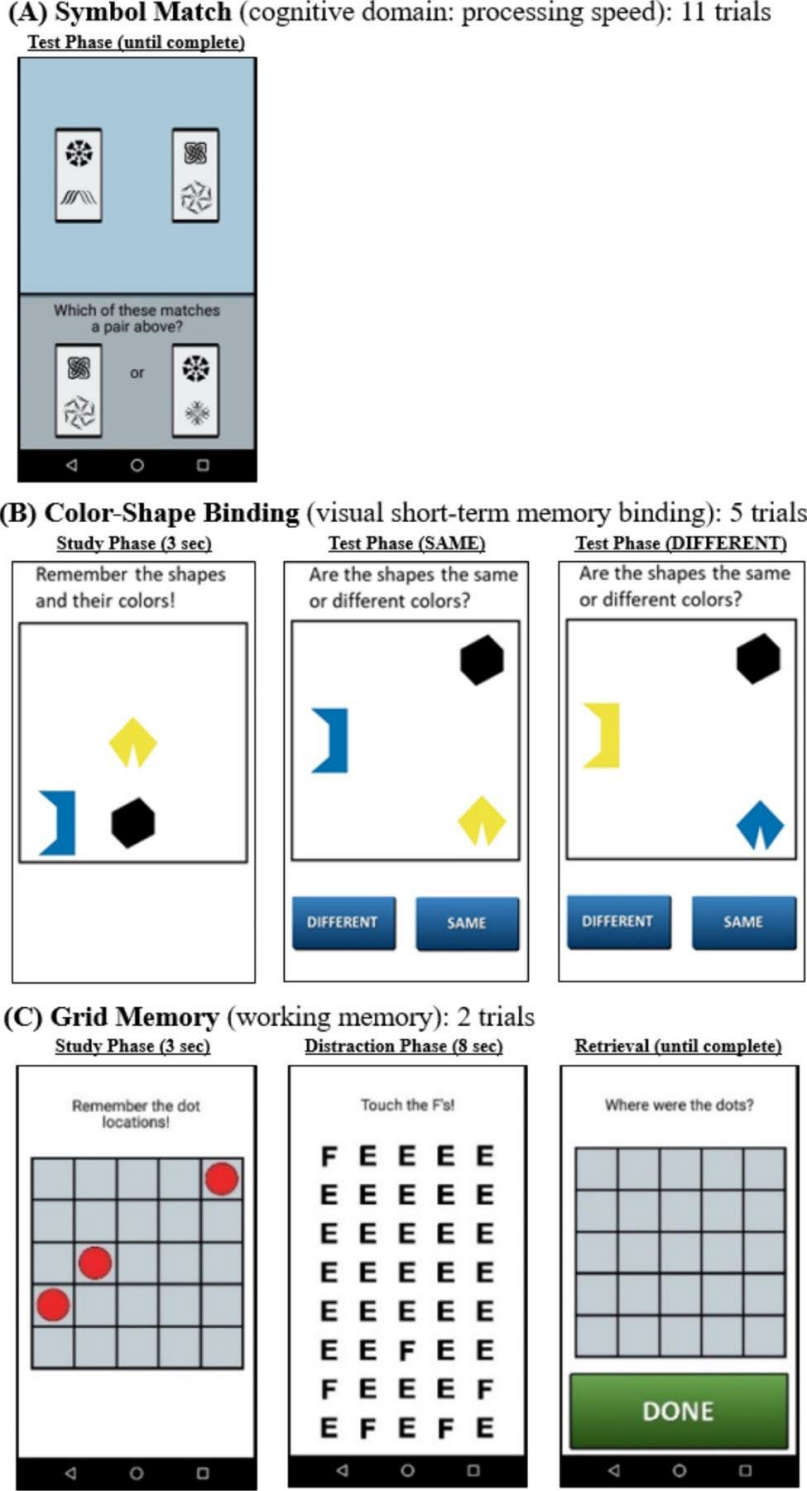
Screenshots of mobile cognitive tasks

The current study used three mobile cognitive tasks to measure cognitive performance (Fig. 1). Symbol Match task was used to measure processing speed. For assessing working memory performance, Color-Shape Binding task was used to measure visual short-term memory binding, and Grid Memory task was used to measure spatial working memory. Each cognitive task took an average of 45 s to complete at each assessment occasion (i.e., session). Reliability and validity of the EMA Symbol Match and Grid Memory task measures [28], as well as preliminary validity evidence for the Color-Shape Binding measure [29], were established in prior research.

#### Symbol match (cognitive domain: processing speed)

Participants were asked to compare two symbol pairs at the top of the screen with two symbol pairs at the bottom of the screen and decide as quickly and accurately as possible which of the bottom-screen pairs matches a top-screen pair. The task comprised of 11 trials. Mean response time (unit: seconds) of correct trials within each assessment occasion was used to operationalize performance. Higher values reflected slower processing speed [28].

#### Color-Shape binding (cognitive domain: visual short-term memory binding)

Participants were asked to memorize the shapes and colors of three different polygons for 3 s. The three polygons were then removed from the screen and re-displayed at different locations, either having the same or different colors. Participants were asked to decide whether the combination of colors and shapes are the “Same” or “Different” between the study and task phases. The task comprised of 5 trials. We used d-prime (i.e., z-scored hit (proportion of correct “Different” responses) – z-scored false alarm (proportion of incorrect “Different” responses)) to indicate correct recognition score. Higher values reflected better binding performance [27].

#### Grid memory (cognitive domain: spatial working memory)

Participants were asked to memorize the location of 3 dots presented at random locations on a 5 × 5 grid for 3 s. After an 8-second distractor phase, where participants were asked to touch all letter E’s among a grid of letter F’s, they were then asked to recall the location of each dot during the study phase. The task comprised of 2 trials. Performance was operationalized as an error score averaged across two trials, i.e., the Euclidean distance between the correct and participant-reported dot locations. Higher error scores reflected worse spatial working memory performance [28, 31].

### Availability of healthy foods

Objective availability of healthy foods was defined as the density of grocery stores and supermarkets (NAICS 445,110) as well as specialty food stores (NAICS 4452). Establishment variables were derived from the National Establishment Time-Series (NETS) database, and all measures were counts per 1000 population in a census track (mean centered). To deal with outliers, scores were top-coded to the 99th-percentile [18]. The data for each census tract was obtained from the National Neighborhood Data Archive database [32].

Subjective availability of healthy foods was assessed using a self-report questionnaire that asked participants the degree to which they agreed/disagreed to the following three items: “A large selection of fresh fruits and vegetables is available in my neighborhood”, “The fresh fruits and vegetables in my neighborhood are of high quality”, and “A large selection of low-fat products is available in my neighborhood”, with responses on a 5-point Likert scale (1 = Strongly disagree, 5 = Strongly agree) [33]. Our sample completed all three items of the measure of subjective availability of healthy foods. The composite variable, averaged across these three items and centered at the sample mean, was used to indicate availability of healthy foods as our main independent variable. We also created tertiles for the availability of healthy foods score (low, intermediate, high availability of healthy foods) to further assess whether the pattern of associations of categorical food environment measures with cognition is similar to the findings for the continuous measures.

### Covariates

The following *demographic and individual-level covariates* were included: *age* was coded in years (centered at the sample mean of 77), *gender* was coded as ‘male (0)’ and ‘female’, and *race/ethnicity* was coded as ‘non-Hispanic Whites’, ‘non-Hispanic Blacks’, and ‘other’. *Education* was measured using the highest degree earned and coded as ‘Less than high school’, ‘High school diploma’, ‘Associate/Bachelor’, and ‘Master/Doctorate’. *Financial situation* was assessed using one item, “How would you rate your financial situation these days?” (0 = Worst possible situation through, 10 = Best possible situation) and centered at the sample mean. *Fruit and vegetable consumption* was assessed using two items from the Rapid Eating and Activity Assessment for Participants Short Version (REAP-S) [34]. These items were, “In an average week, how often do you eat more than 2 servings of fruit a day? (serving = ½ cup or 1 medium fruit or ¾ cup 100% fruit juice)” and “eat more than 2 servings of vegetables a day? (serving = ½ cup vegetables or 1 cup leafy raw vegetables)” (1 = Rarely/never, 2 = Sometimes, 3 = Usually/often). A summary variable was calculated as the average of two items and centered at 2 (Sometimes). History of *diabetes* (0 = no, 1 = yes) and *high blood pressure* (0 = no, 1 = yes), and *average daily walking minutes* were assessed through self-reports at baseline. *MCI* was defined using the Jak/Bondi actuarial criteria [35]. Five cognitive domains (memory, executive function, attention, language, and visual-spatial) from ten neuropsychological instruments were used for the MCI classification. Each domain included two neuropsychological instruments (see [30] for details). The following actuarial formula was used: (i) impaired scores, defined as 1 SD below the age, gender, and education adjusted normative mean, on both measures within at least one cognitive domain; or (ii) impaired scores, defined as 1 SD below the age, gender, and education adjusted normative mean, in three neuropsychological instruments among five cognitive domains. If neither of these criteria was met, a score of 4, indicating the number of items of functional inability on all four instrumental activities of daily activities items on the Lawton Brody scale [36] must occur for an individual to be classified as MCI.

*Subjective neighborhood quality*. *Perceived safety* was measured using three items: “I feel safe walking in my neighborhood at night”, “Violence is not a problem in my neighborhood”, and “My neighborhood is safe from crime” (1 = Strongly disagree, 5 = Strongly agree). *Perceived aesthetic quality* was measured using five items including: “There is a lot of trash and litter in my neighborhood (reverse coded)”, “There is a lot of noise in my neighborhood (reverse coded)”, “In my neighborhood the buildings and homes are well-maintained”, “The buildings and houses in my neighborhood are interesting”, and “My neighborhood is attractive”. *Perceived social cohesion* was measured using the four items including: “People in my neighborhood generally get along with each other”, “People around here are willing to help their neighbors”, “People in my neighborhood can be trusted”, and “People in my neighborhood share the same values”. Summary variables for safety, aesthetic quality, and social cohesion were calculated as the average of items in each domain respectively. These measures were controlled for to confirm the effect of perceived availability of healthy foods over and above the potential response style bias from self-reports (i.e., a tendency to use the rating scale in a certain systematic way regardless of actual content).

To indicate *neighborhood deprivation*, publicly available *Area Deprivation Index (ADI)* score was used, which is a factor-based index that uses 17 US Census–based poverty, education, housing quality, and employment indicators to characterize and rank the socioeconomic contextual disadvantage of a particular neighborhood at the census block group level. Participants’ most recently reported address was linked to ADI state decile score (1 = Least deprived to 10 = Most deprived) through 12-digit Federal Information Processing System (FIPS) code received from the Census geocoding API and centered at the sample mean. For the *assessment-level covariates* to model retest effects and time of day effects on EMA cognitive tasks, *session number* (i.e., session 1, 2, 3, 4…) and *time of day* were used respectively.

### Analytic approach

To examine whether food environments were associated with cognition, two level multilevel mixed models (MLMs) were estimated in SAS PROC MIXED (version 9.4). The use of MLMs allowed us to account for the nested structure of the data (i.e., assessments within persons) and to estimate cognitive performance precisely after controlling for retest (from repeated testing) and time of day effects. Full maximum likelihood was used for model estimation and robust standard errors were used for fixed effects hypothesis testing. Each cognitive task score (i.e., Symbol Match, Color-Shape Binding, and Grid Memory) was modeled as a function of availability of healthy foods in separate models. Covariates were age, sex, race/ethnicity, education, financial situation, ADI, retest effects, and time of day effects. Shown below is the multilevel model for the effect of availability of healthy foods on Symbol Match task performance (i.e., response time) that specifies two levels of analysis.

Level 1:

$$\begin{array}{l}Response{\mkern 1mu} tim{e_{ij}} = {b_{0j}} + {b_{1j}}\left( {Sessio{n_{ij}}} \right)\\+ {b_{2j}}\left( {Sessio{n_{ij}} \times Sessio{n_{ij}}} \right)\\+ {b_{3j}}\left( {Time{\mkern 1mu} of{\mkern 1mu} da{y_{ij}}} \right)\\+ {b_{4j}}\left( {Time{\mkern 1mu} of{\mkern 1mu} da{y_{ij}} \times Time{\mkern 1mu} of{\mkern 1mu} da{y_{ij}}} \right) + {e_{ij}}\end{array}$$

The Level 1 model describes within-person variation in response time of the Symbol Match task for person j on assessment i as a function of a person-specific intercept (${b}_{0j}$), linear and quadratic retest effect (${b}_{1j}, {b}_{2j}$), linear and quadratic time of day effect (${b}_{3j}, {b}_{4j}$), and an assessment- and person-specific residual deviation from that intercept (${e}_{ij}$).

Level 2:

$$\begin{array}{l}{b_{0j}} = {\beta _{00}}\\+ {\beta _{01}}\left( {Objective\,availability\,of\,healthy\,food{s_{.j}}} \right)\\+ {\beta _{02}}\left( {Subjective\,availability\,of\,healthy\,food{s_{.j}}} \right)\\+ {\beta _{03}}\left( {Ag{e_{.j}}} \right) + {\beta _{04}}\left( {Se{x_{.j}}} \right) + {\beta _{05}}\left( {Rac{e_{.j}}} \right)\\+ {\beta _{06}}\left( {Educatio{n_{.j}}} \right) + {\beta _{07}}\left( {Financia{l_{.j}}} \right)\\+ {\beta _{08}}\left( {AD{I_{.j}}} \right) + {u_{0j}}\end{array}$$

$${b}_{1j}= {\beta }_{10}+{u}_{1j}$$

$${b}_{2j}= {\beta }_{20}$$

$${b}_{3j}= {\beta }_{30}+{u}_{2j}$$

$${b}_{4j}= {\beta }_{40}$$

The Level 2 model describes between-person variation in the mean response time across entire assessments. ${\beta }_{00}$ represents the sample average response time for 77-year-old, non-Hispanic White men with high school diploma who had average ratings financial situation and ADI score. ${\beta }_{01}$ and ${\beta }_{02}$ reflect the differences in response time with a 1 unit between-person difference in objective (for ${\beta }_{01}$) or subjective (for ${\beta }_{02}$) availability of healthy foods. ${\beta }_{03}$ indicates the difference in response time with a 1-year difference in age, ${\beta }_{04}$ indicates sex differences in response time, ${\beta }_{05}$ indicates racial differences in response time, ${\beta }_{06}$ indicates educational differences in response time, and ${\beta }_{07}$ indicates differences in response time by financial situation, and ${\beta }_{08}$ indicates ADI differences in response time. ${\beta }_{10}$ and ${\beta }_{20}$ indicate linear and quadratic retest effect, and ${\beta }_{30}$ and ${\beta }_{40}$ indicate linear and quadratic time of day effect. Finally, ${u}_{0j}$, ${u}_{1j}$, and ${u}_{2j}$ reflect person-specific deviations from the average level of response time, retest effects, and time of day effects respectively. Our interest was to examine ${\beta }_{01}$ and ${\beta }_{02}$, the average effects of objective and subjective availability of healthy foods, after controlling for covariates. Identical models were fitted for the other two cognitive outcomes.

Next, we examined whether behavioral and cardiovascular factors (i.e., *M*) mediated the association between availability of healthy foods (i.e., *X*) and cognition (i.e., *Y*). As the analysis involved upper-level mediation, i.e., the effect of a Level 2 predictor (availability of healthy foods) on a Level 1 outcome (cognition) is mediated by another Level 2 predictor (e.g., fruit and vegetable consumption, walking, hypertension and diabetes), we conducted simple mediation analysis [37]. To calculate the indirect effect, we first calculated the coefficient (*a*) for *X* in a model predicting *M*. Then we calculated the coefficient (*b*) for *M* predicting *Y* after controlling for *X*. The product of *a* and *b* indicates the indirect effect of *X* on *Y* through *M*. To build confidence intervals around the indirect effect, a Monte Carlo simulation with 20,000 replications was used from the method proposed by Selig and Preacher [38].

## Results

### Descriptive statistics

Mean age was 77.5 (SD = 4.8) and women made up 67.6% of the sample. The sample was diverse in terms of race (non-Hispanic White: 45.7%, non-Hispanic Black: 40.3%, other: 14.0%) and education (Less than high school: 5.7%, High school or GED: 43.2%, Associates/Bachelors: 26.0%, and Masters/Doctorate: 25.1%). 31% of the sample had mild cognitive impairment (MCI); high prevalence of MCI may be due to diverse sample characteristics (e.g., 40% of non-Hispanic Black). The sample mean of objective availability of healthy foods was 1.1 between 0 and 5.1 (for the tertile measure, low availability = 0 to 0.5, intermediate = 0.5 to 1.1, high = 1.1 to 5.1). The sample mean of subjective availability of healthy foods was 3.9 between 1 and 5 (for the tertile measure, low availability = 1 to 3.3, intermediate = 3.7 to 4.0, high = 4.3 to 5.0). The mean ADI score was 5.8 between 1 (least deprived) and 10 (most deprived). The sample mean for their perceived financial situation was 7.1 between 0 (Worst) and 10 (Best), and the mean fruit and vegetable consumption was 2.3, representing average response of “Sometimes”. Daily average walking duration was 19 min. 67% of the sample had a history of hypertension, and 23% of the sample had a history of diabetes. The average number of EMA sessions completed was 70 (range = 14 to 97) (Table 1).

**Table 1:** Descriptive statistics (N = 315)

Variable	Mean (SD) or %	Range
Age	77.46 (4.84)	70 to 91
Female	67.62%	-
Race		
Non-Hispanic Whites	45.71%	-
Non-Hispanic Blacks	40.32%	-
Other race	13.97%	-
Years of education		
Below high school completion	5.71%	-
High school diploma/GED	43.17%	-
Associates/Bachelors	26.03%	-
Masters/ Doctorate	25.08%	-
Objective availability of healthy foods	1.09 (0.99)	0 to 5.12
Subjective availability of healthy foods	3.93 (0.83)	1 to 5
Area Deprivation Index	5.81 (2.29)	1 to 10
Financial situation	7.05 (2.18)	0 to 10
Fruit and vegetable consumption	2.25 (0.63)	1 to 3
Daily walking minutes	19.19 (35.43)	0 to 240
History of hypertension	66.88%	
History of diabetes	23.25%	
Number of EMA sessions completed	70.17 (15.25)	14 to 97
Symbol Match (seconds) ^a,b^	3.25 (0.89)	1.27 to 6.91
Color-Shape Binding (d prime) ^a,c^	0.63 (0.29)	-0.06 to 0.99
Grid Memory (Euclidean error distance) ^a,d^	2.25 (0.85)	0.12 to 4.26

^a^ Measures are aggregated across all sessions for each person.

^b^ Unit: Response time in seconds. Higher scores mean low cognitive function.

^c^ Unit: d prime (z(H) - z(F)). Higher scores mean better cognitive function (more accurate responses with fewer false alarms).

^d^ Unit: Euclidean error distance. Higher scores mean worse cognitive function.

Results from the correlation analysis (Table 2) suggested that greater objective availability of healthy foods was correlated with worse subjective availability of healthy foods (r = ‒.13), less neighborhood-level deprivation (r = ‒.15), and worse financial situation (r = ‒.12), but was not correlated with any cognitive measures. Greater subjective availability of healthy foods was correlated with better financial situation (r = .25), more fruit and vegetable consumption (r = .19), and better cognition (r = ‒.20 for Symbol Match, r = .17 for Color-Shape Binding, and r = ‒.18 for Grid Memory). Fruit and vegetable consumption was correlated with better cognition (r = ‒.21 for Symbol Match, r = .27 for Color-Shape Binding, and r = ‒.20 for Grid Memory). More daily walking was correlated with better scores in Symbol Match task (r = ‒.12).

**Table 2:** Correlations among key variables

	1	2	3	4	5	6	7	8
1. Objective availability of healthy foods	-							
2. Subjective availability of healthy foods	-0.13^*^	-						
3. Area Deprivation Index	-0.15^*^	-0.09	-					
4. Financial situation	-0.12^*^	0.25^**^	0.00	-				
5. Fruit and vegetable consumption	-0.03	0.19^**^	-0.06	0.20^**^	-			
6. Daily walking minutes	-0.02	0.03	-0.01	0.12^*^	0.08	-		
7. Symbol Match	-0.02	-0.20^**^	0.10	-0.11^*^	-0.21^**^	-0.12^*^	-	
8. Color-Shape Binding	0.01	0.17^**^	-0.01	0.16^**^	0.27^**^	0.05	-0.53^**^	-
9. Grid Memory	-0.01	-0.18^**^	0.14^*^	-0.12^*^	-0.20^**^	0.05	0.33^**^	-0.52^**^

*Note.* Spearman correlation analyses were conducted; ^*^ p < .05, ^**^ p < .01

### Association between availability of healthy foods and cognition

We examined whether objective and subjective measures of food environment were associated with better cognitive performance in different tasks (i.e., Symbol Match, Color-Shape Binding, and Grid Memory). For *Symbol Match* task performance (cognitive domain: processing speed), results from the multilevel models (Table 3) indicated that older age, being non-Hispanic Black vs. non-Hispanic White, and not having a high school degree vs. having a high school degree were significantly associated with slower response times (RT). The linear and quadratic retest effects (i.e., session number) were significant, indicating that RT improved over time. The linear and quadratic time of day effects were also significant, indicating that RT slowed throughout the day. The effect of objective availability of healthy foods was not significant. The effect of subjective availability of healthy foods was significant: a one-point increase in the perceived availability was associated with 176 milliseconds faster RT after controlling for objective availability and other covariates. For *Color-Shape Binding* task performance (domain: visual short-term memory binding), results from the multilevel models (Table 4) indicated that higher scores in subjective availability of healthy food availability were associated with more accurate responses (estimate = 0.042) after controlling for objective food environment measure and covariates. Objective availability of healthy foods was not associated with Color-Shape Binding task performance. There was no significant association of either objective or subjective availability of healthy foods with *Grid Memory* task performance (domain: spatial working memory) (Table 5). We also conducted the above analyses separately for objective and subjective availability of healthy foods, and the pattern of results was similar (Supplementary Table 1).

**Table 3:** Results from multilevel models (outcome: Symbol Match (processing speed))

Fixed effects	Estimate (SE)	95% CI	p-value
Intercept	3.362 (0.200)	[2.968, 3.757]	< 0.001
Objective availability of healthy foods	-0.063 (0.045)	[-0.151, 0.025]	0.159
Subjective availability of healthy foods	-0.176 (0.060)	[-0.293, -0.059]	0.003
Linear session	-0.008 (0.001)	[-0.010, -0.006]	< 0.001
Quadratic session	0.000 (0.000)	[0.000, 0.000]	< 0.001
Linear time of day	0.006 (0.001)	[0.004, 0.008]	< 0.001
Quadratic time of day	0.002 (0.000)	[0.001, 0.002]	< 0.001
Age	0.033 (0.011)	[0.012, 0.055]	0.003
Female	-0.138 (0.107)	[-0.347, 0.071]	0.197
nHB^a^ vs. nHW^b^	0.265 (0.121)	[0.028, 0.503]	0.029
Other race vs. nHW^b^	0.239 (0.139)	[-0.034, 0.513]	0.086
Below HS vs. HS^c^	0.685 (0.250)	[0.195, 1.175]	0.006
Associates/Bachelors vs. HS	-0.034 (0.118)	[-0.264, 0.197]	0.775
Graduate vs. HS	0.072 (0.135)	[-0.193, 0.337]	0.595
Financial situation	-0.011 (0.025)	[-0.061, 0.038]	0.653
ADI^d^	0.010 (0.021)	[-0.031, 0.051]	0.640
**Random effects**			
Var (Intercept)	0.664 (0.059)		< 0.001
Var (Session)	0.000 (0.000)		< 0.001
Var (Time)	0.000 (0.000)		< 0.001
Covar (Intercept, Session)	-0.001 (0.000)		0.000
Covar (Intercept, Time)	0.001 (0.001)		0.397
Covar (Session, Time)	0.000 (0.000)		0.642
Residual	0.283 (0.003)		< 0.001

*Note.* Unit: Response time in seconds. Higher scores mean low cognitive function.

^a^ nHB = non-Hispanic Blacks; ^b^ nHW = non-Hispanic Whites; ^c^ HS = High school completion;

^d^ Area Deprivation Index

**Table 4:** Results from multilevel models (outcome: Color-Shape Binding (visual short-term memory binding))

Fixed effects	Estimate (SE)	95% CI	p-value
Intercept	0.547 (0.062)	[0.425, 0.669]	< 0.001
Objective availability of healthy foods	0.025 (0.015)	[-0.004, 0.055]	0.094
Subjective availability of healthy foods	0.042 (0.017)	[0.009, 0.075]	0.012
Linear session	0.004 (0.001)	[0.003, 0.005]	< 0.001
Quadratic session	0.000 (0.000)	[0.000, 0.000]	< 0.001
Linear time of day	-0.001 (0.000)	[-0.001, 0.000]	0.094
Quadratic time of day	0.000 (0.000)	[0.000, 0.000]	0.618
Age	-0.009 (0.004)	[-0.016, -0.001]	0.023
Female	0.023 (0.036)	[-0.046, 0.093]	0.510
nHB^a^ vs. nHW^b^	-0.129 (0.042)	[-0.211, -0.046]	0.002
Other race vs. nHW^b^	-0.046 (0.05)	[-0.144, 0.051]	0.350
Below HS vs. HS^c^	-0.218 (0.08)	[-0.374, -0.062]	0.006
Associates/Bachelors vs. HS	0.102 (0.041)	[0.022, 0.183]	0.013
Graduate vs. HS	0.117 (0.039)	[0.040, 0.195]	0.003
Financial situation	-0.002 (0.007)	[-0.015, 0.012]	0.819
ADI^d^	0.015 (0.007)	[0.001, 0.029]	0.040
**Random effects**			
Var (Intercept)	0.063 (0.006)		< 0.001
Residual	0.085 (0.001)		< 0.001

*Note.* Unit: d prime (z(H) - z(F)). Higher scores mean better cognitive function (more accurate responses with fewer false alarms).

^a^ nHB = non-Hispanic Blacks; ^b^ nHW = non-Hispanic Whites; ^c^HS = High school completion;

^d^ Area Deprivation Index

**Table 5:** Results from multilevel models (Outcome: Grid Memory (spatial working memory))

Fixed effects	Estimate (SE)	95% CI	p-value
Intercept	2.275 (0.177)	[1.928, 2.623]	< 0.001
Objective availability of healthy foods	-0.094 (0.051)	[-0.194, 0.006]	0.065
Subjective availability of healthy foods	-0.079 (0.051)	[-0.179, 0.022]	0.125
Linear session	-0.005 (0.002)	[-0.008, -0.002]	0.003
Quadratic session	0.000 (0.000)	[0.000, 0.000]	0.719
Linear time of day	0.004 (0.001)	[0.001, 0.007]	0.003
Quadratic time of day	0.001 (0.000)	[0.001, 0.001]	< 0.001
Age	-0.001 (0.009)	[-0.018, 0.016]	0.899
Female	0.412 (0.101)	[0.215, 0.609]	< 0.001
nHB^a^ vs. nHW^b^	0.248 (0.106)	[0.040, 0.457]	0.020
Other race vs. nHW^b^	0.16 (0.135)	[-0.104, 0.425]	0.236
Below HS vs. HS^c^	0.193 (0.152)	[-0.104, 0.49]	0.203
Associates/Bachelors vs. HS	-0.471 (0.108)	[-0.683, -0.259]	< 0.001
Graduate vs. HS	-0.586 (0.122)	[-0.825, -0.348]	< 0.001
Financial situation	-0.001 (0.021)	[-0.043, 0.041]	0.958
ADI^d^	0.026 (0.020)	[-0.013, 0.065]	0.185
**Random effects**			
Var (Intercept)	0.498 (0.045)		< 0.001
Residual	1.034 (0.011)		< 0.001

*Note.* Unit: Euclidean error distance. Higher scores mean worse cognitive function.

^a^ nHB = non-Hispanic Blacks; ^b^ nHW = non-Hispanic Whites; ^c^HS = High school completion;

^d^ Area Deprivation Index

We ran several sensitivity analyses. First, to rule out the reverse causality concern, we repeated main analyses after removing individuals with MCI. The significant effects of subjective availability of healthy foods stayed similar after removing those individuals (estimate= -0.181, 95% CI=[-0.296, -0.066] for Symbol Match; estimate = 0.050, 95% CI=[0.015, 0.086] for Color-Shape Binding). Second, we examined whether the effects of subjective food environments would be stronger among potential food preparers (i.e., individuals who live alone vs. not; individuals who plans/prepares/serves meals independently vs. not). We did not find moderation effects of living alone or meal preparation in the associations. Third, to rule out the possible biases from self-reports, we controlled for other perceived neighborhood quality measures (i.e., perceived safety, aesthetic quality, and social cohesion) in separate models. The pattern of results stayed similar especially for Symbol Match. Fourth, we used tertiles of the availability of healthy foods (low, intermediate, and high) to further assess the pattern of the association with cognitive performance. For subjective availability of healthy foods, high versus low availability was associated with better cognition (estimate= -0.308, 95% CI=[-0.567, -0.049] for Symbol Match; estimate = 0.106, 95% CI=[0.028, 0.183] for Color Shape). Individuals with high versus intermediate availability showed better cognition (estimate= -0.212, 95% CI=[-0.441, 0.017] for Symbol Match; estimate = 0.085, 95% CI=[0.015, 0.155] for Color Shape). There were no differences between intermediate versus low subjective availability of healthy foods. For objective availability of healthy foods, there were no significant differences in cognition among high, intermediate, and low availability.

### Pathways in the association between availability of healthy foods and cognition

Next, we conducted mediation analyses to evaluate the mechanisms through which subjective availability of healthy foods was associated with cognition. The indirect effects for fruit and vegetable consumption were significant (for Symbol Match, indirect effect = -0.025, CI=[-0.0569, -0.0021], percent mediated = 13.9%; for Color-Shape Binding: indirect effect = 0.007, CI=[0.0002, 0.0162], percent mediated = 15.8%), indicating that fruit and vegetable consumption partially mediated the association between subjective availability of healthy foods and cognitive performance. Average daily walking minutes or history of hypertension and diabetes did not mediate the associations between subjective food environments and cognition. In addition, there were no significant mediating effects for objective food environments.

## Discussion

Despite growing evidence for the importance of neighborhood characteristics for late-life cognitive health, few studies have examined the associations between food environments and cognitive health. In the current study, we found that greater subjective availability of healthy foods was associated with better processing speed and memory binding performance. The associations persisted after accounting for objective food environment measure, individual-level socio-demographic factors and neighborhood deprivation; objective food environment measure was not associated with cognition. We also found that 14 to 16% of effects of subjective availability of healthy foods on cognition were mediated through fruit and vegetable consumption. Given that processing speed and memory binding performance measures reflect cognitive domains sensitive to age-related deficits and early marker of disease, our results suggest that the subjective availability of healthy foods may be associated with cognitive deficits in early stages of cognitive aging and/or impairment. That is, the observed cognitive differences could arise from acceleration of normative age-related mechanisms or from an increased risk of non-normative pathological mechanisms.

Importantly, subjective rather than objective availability of healthy foods was associated with cognition. Objective measures may not capture important non-geographic dimensions of food environments regarding individuals’ experiences that may be influenced by various factors (e.g., transportation, local food stores’ business strategies, staff and service) [12, 39]. In contrast, self-reported perceptions of immediate food environment likely reflect the actual use and experiences of local food stores, and thus may be a more proximal factor for health-related behaviors and cognitive health outcomes. Relatedly, prior research found that subjective measures of healthy food availability were consistently related to multiple healthy dietary outcomes, but objective measures were overwhelmingly unrelated to dietary outcomes [8].

We also found partial but significant mediating effects of fruit and vegetable consumption in the associations between subjective availability of healthy foods and cognition. Although prior studies [21, 22] have found the protective role of fruit and vegetable consumption in late-life cognitive health, little is known about how these protective behaviors are contextualized in the individuals’ built environment. This study extends prior research by emphasizing the role of food environments that may shape healthy dietary behaviors influencing cognitive health. Given that health promoting behaviors cannot be sustained without supportive environmental contexts, the current finding has implications for sustainable behavioral interventions to promote cognitive health.

There may be other mechanisms through which subjective food environments are associated with late-life cognition, which require future investigation. One possibility is that better subjective food environments are associated with opportunities for mental, social, and physical activities. In urban areas, older adults are likely to purchase food in small batches, and shop at multiple stores to obtain quality foods (Munoz-Plaza et al., 2013). Thus, urban older adults who perceive greater availability of healthy foods may go out and shop frequently to get fresh and good quality foods in their neighborhood. Not being distracted along the way to the supermarkets, thinking about the shopping list, choosing among various items may serve as a brain training that happens several times a week [17]. Moreover, food shopping can provide older adults with a chance of exercise as well as a source of social interaction [40, 41], which in turn may promote cognitive health.

Another possibility is that greater availability of healthy foods may be a proxy for the availability of other institutional resources, which in turn can provide perceptual/cognitive stimulation and activate neural networks related to alertness, sustained attention, and response to novelty [42]. Greater perceived availability of healthy food stores may motivate older adults to use other environmental facilities (e.g., libraries, community centers) on the similar walking paths, which all facilitate mental stimulation (e.g., reading books, social interactions) [43, 44]. It may also be the case that older adults living in the neighborhood with better institutional resources have a lifelong history of living in more resource-rich environments, which might have helped them accumulate cognitive reserve [43]. Alternatively, perceived availability of healthy foods may reflect neighborhood-level socioeconomic status. Prior studies found that living in deprived neighborhoods had a moderately strong negative association with cognition [5], possibly due to lower availability of healthy food stores or walking paths. However, the effect of subjective availability of healthy foods was significant after controlling for neighborhood deprivation.

The findings from this study have several implications for future research and policy. First, as subjective food environment measures may provide additional information not captured by objective measures, future studies may need to include both subjective and objective food environment measures to identify features of food environments linked to cognitive health. In addition, studies should identify factors influencing individuals’ perceptions of local food environments (e.g., cultural habits, demographic and psychological characteristics). Second, given that changes in objective food environment alone (e.g., introducing new retailers) may not be sufficient to promote health-related behaviors [45], policy changes and interventions will need to utilize subjective food environment measures to help decide the design of interventions and to evaluate the effectiveness of interventions.

This study has several limitations. First, this study is cross-sectional in nature, and the direction of causal relationship remains uncertain. It is possible that older adults with better mental and physical health go out and navigate their neighborhood frequently in their daily lives and perceive their food environments more favorably. Then poorer perception of healthy foods availability may be a proxy for early stage of cognitive or functional impairment. However, after excluding individuals having MCI, the patterns of results stayed similar. Second, various measures to assess food environments (e.g., affordability of healthy foods, different types of food stores) [8, 46, 47] would be required in future studies to fully understand the impact of local food environments. Third, although family support related to food shopping and preparation may contribute to an individual’s perceptions and experiences of local food options, we did not have the measures. Future studies will need to address the influence of family support on individuals’ perception of food environments. Fourth, we could not examine whether better perceived availability of healthy foods is associated with more frequent shopping and going out. The use of person-specific, GPS-based activity space [48–50], accurate measures of physical activity using digital technology, and smartphone-administered EMA cognition measures will provide high resolution spatio-temporal data and allow us to examine precise behavioral mechanisms through which neighborhood food environments are associated with older adults’ cognitive health. By identifying precise behavioral-level mechanisms through which contexts are associated with cognitive health, we will be able to design population- and individual-level interventions to promote cognitive health.

## Conclusion

Subjective availability of healthy foods was associated with processing speed and memory binding performance among urban, community dwelling older adults. The effects of the subjective availability of healthy foods were significant after accounting for objective food environments, demographic variables, and individual- and neighborhood-level socioeconomic status. Fruit and vegetable consumption partially mediated associations between subjective availability of healthy foods and cognition. These findings suggest the importance of local food environments in shaping individuals’ health-related behaviors, which in turn may influence cognitive health. The findings further suggest the utility of subjective food environment measures in future studies and interventions. Subjective measures may reflect individuals’ actual experiences with local food environments not captured by objective measures. Thus, it would be important to include both objective and subjective food environment measures in identifying impactful target for intervention and evaluating effectiveness of policy changes.

## Electronic supplementary material

Below is the link to the electronic supplementary material.

Supplementary Material 1

## Data Availability

The datasets generated and/or analyzed during the current study are available from the corresponding author after obtaining approval from the Einstein Aging Study Scientific Committee.

## List of Abbreviations

EMA: Ecological momentary assessment
EAS: Einstein Aging Study
MCI: Mild cognitive impairment

## References

[1] Alzheimer’s Association. 2022 Alzheimer’s Disease Facts and Figures. Chicago, IL: Alzheimer’s Association; 2022.

[2] Lipnicki DM, Makkar SR, Crawford JD, Thalamuthu A, Kochan NA, Lima-Costa MF (2019). Determinants of cognitive performance and decline in 20 diverse ethno-regional groups: a COSMIC collaboration cohort study. PLoS Med.

[3] Livingston G, Huntley J, Sommerlad A, Ames D, Ballard C, Banerjee S (2020). Dementia prevention, intervention, and care: 2020 report of the Lancet Commission. The Lancet.

[4] Besser LM (2021). Outdoor green space exposure and brain health measures related to Alzheimer’s disease: a rapid review. BMJ open.

[5] Besser LM, McDonald NC, Song Y, Kukull WA, Rodriguez DA (2017). Neighborhood environment and cognition in older adults: a systematic review. Am J Prev Med.

[6] Chen X, Lee C, Huang H (2022). Neighborhood built environment associated with cognition and dementia risk among older adults: a systematic literature review. Soc Sci Med.

[7] Wu YT, Prina AM, Brayne C. The association between community environment and cognitive function: A systematic review. Soc Psychiatry Psychiatr Epidemiol. 2015 Mar 1;50(3):351–62. 10.1007/s00127-014-0945-6 PMC4322216 25087013

[8] Caspi CE, Sorensen G, Subramanian SV, Kawachi I (2012). The local food environment and diet: a systematic review. Health Place.

[9] Morland KB, Evenson KR (2009). Obesity prevalence and the local food environment. Health Place.

[10] Vadiveloo MK, Sotos-Prieto M, Parker HW, Yao Q, Thorndike AN (2021). Contributions of food environments to dietary quality and cardiovascular disease risk. Curr Atheroscler Rep.

[11] Coulton CJ, Jennings MZ, Chan T (2013). How big is my neighborhood? Individual and contextual effects on perceptions of neighborhood scale. Am J Community Psychol.

[12] Munoz-Plaza CE, Morland KB, Pierre JA, Spark A, Filomena SE, Noyes P (2013). Navigating the urban food environment: challenges and resilience of community-dwelling older adults. J Nutr Educ Behav.

[13] Gajda R, Jeżewska-Zychowicz M (2020). Elderly perception of distance to the grocery store as a reason for feeling food insecurity—can food policy limit this?. Nutrients.

[14] Roosa MW, White RM, Zeiders KH, Tein JY (2009). An examination of the role of perceptions in neighborhood research. J Community Psychol.

[15] Laméris J, Hipp JR, Tolsma J (2018). Perceptions as the crucial link? The mediating role of neighborhood perceptions in the relationship between the neighborhood context and neighborhood cohesion. Soc Sci Res.

[16] Fangfang H, Xiao H, Shuai Z, Qiong W, Jingya Z, Guodong S (2022). Living environment, built environment and cognitive function among older chinese adults: results from a cross-sectional study. J Prev Alzheimer’s Disease.

[17] Tani Y, Suzuki N, Fujiwara T, Hanazato M, Kondo K (2019). Neighborhood food environment and dementia incidence: the japan gerontological evaluation study cohort survey. Am J Prev Med.

[18] Finlay J, Esposito M, Langa KM, Judd S, Clarke P (2022). Cognability: an ecological theory of neighborhoods and cognitive aging. Soc Sci Med.

[19] Sallis JF, Cervero RB, Ascher W, Henderson KA, Kraft MK, Kerr J (2006). An ecological approach to creating active living communities. Annu Rev Public Health.

[20] Glass TA, McAtee MJ (2006). Behavioral science at the crossroads in public health: extending horizons, envisioning the future. Soc Sci Med.

[21] Loef M, Walach H (2012). Fruit, vegetables and prevention of cognitive decline or dementia: a systematic review of cohort studies. J Nutr Health Aging.

[22] Mottaghi T, Amirabdollahian F, Haghighatdoost F (2018). Fruit and vegetable intake and cognitive impairment: a systematic review and meta-analysis of observational studies. Eur J Clin Nutr.

[23] Deary I (2000). Looking down on Human Intelligence: from psychometrics to the brain.

[24] Madden DJ, Birren JE, Schaie KW (2001). Speed and timing of behavioral processes. Handbook of the psychology of aging.

[25] Salthouse TA (1996). The processing-speed theory of adult age differences in cognition. Psychol Rev.

[26] Reuter-Lorenz PA, Sylvester CYC, Cabeza R, Nyberg L, Park DC (2004). The cognitive neuroscience of working memory and aging. Cognitive neuroscience of aging.

[27] Parra MA, Abrahams S, Logie RH, Méndez LG, Lopera F, Della Sala S (2010). Visual short-term memory binding deficits in familial Alzheimer’s disease. Brain.

[28] Sliwinski MJ, Mogle JA, Hyun J, Munoz E, Smyth JM, Lipton RB (2018). Reliability and validity of ambulatory cognitive assessments. Assessment.

[29] Cerino ES, Katz MJ, Wang C, Qin J, Gao Q, Hyun J et al. Variability in cognitive performance on mobile devices is sensitive to mild cognitive impairment: Results from the Einstein Aging Study. Front Digit Health. 2021;3. 10.3389/fdgth.2021.758031 PMC8677835 34927132

[30] Katz MJ, Wang C, Nester CO, Derby CA, Zimmerman ME, Lipton RB (2021). T-MoCA: a valid phone screen for cognitive impairment in diverse community samples. Alzheimer’s & Dementia: Diagnosis Assessment & Disease Monitoring.

[31] Siedlecki KL (2007). Investigating the structure and age invariance of episodic memory across the adult lifespan. Psychol Aging.

[32] Finlay J, Mao L, Esposito M, Gomez-Lopez I, Khan A, Clarke P et al. National Neighborhood Data Archive (NaNDA): Grocery Stores by Census Tract, United States, 2003–2017 [Internet]. Ann Arbor, MI: Inter-university Consortium for Political and Social Research [distributor]; 2020. Available from: 10.3886/E123001V1

[33] Mujahid MS, Diez Roux AV, Morenoff JD, Raghunathan T (2007). Assessing the measurement properties of neighborhood scales: from psychometrics to ecometrics. Am J Epidemiol.

[34] Segal-Isaacson CJ, Wylie-Rosett J, Gans KM (2004). Validation of a short dietary assessment questionnaire: the Rapid Eating and Activity Assessment for participants short version (REAP-S). Diabetes Educ.

[35] Bondi MW, Edmonds EC, Jak AJ, Clark LR, Delano-Wood L, McDonald CR (2014). Neuropsychological criteria for mild cognitive impairment improves diagnostic precision, biomarker associations, and progression rates. J Alzheimer’s Disease.

[36] Lawton MP, Brody EM (1970). Assessment of older people: self-maintaining and instrumental activities of daily living. Nurs Res.

[37] Hayes AF (2009). Beyond Baron and Kenny: statistical mediation analysis in the new millennium. Communication Monogr.

[38] Selig JP, Preacher KJ. Monte Carlo method for assessing mediation: An interactive tool for creating confidence intervals for indirect effects [Computer software] [Internet]. 2008. Available from: Available from http://quantpsy.org/

[39] Lesakova D (2016). Seniors and their food shopping behavior: an empirical analysis. Procedia-Social and Behavioral Sciences.

[40] Wilson LC, Alexander A, Lumbers M (2004). Food access and dietary variety among older people. Int J Retail Distribution Manage.

[41] Davis MG, Fox KR, Hillsdon M, Coulson JC, Sharp DJ, Stathi A (2011). Getting out and about in older adults: the nature of daily trips and their association with objectively assessed physical activity. Int J Behav Nutr Phys Activity.

[42] Cassarino M, Setti A (2015). Environment as ‘Brain Training’: a review of geographical and physical environmental influences on cognitive ageing. Ageing Res Rev.

[43] Clarke PJ, Ailshire JA, House JS, Morenoff JD, King K, Melendez R et al. Cognitive function in the community setting: The neighbourhood as a source of ‘cognitive reserve’? J Epidemiol Community Health. 2012 Aug 1;66(8):730–6. 10.1136/jech.2010.128116 PMC3387518 21515547

[44] Ng TP, Nyunt MSZ, Shuvo FK, Eng JY, Yap KB, Hee LM (2018). The neighborhood built environment and cognitive function of older persons: results from the Singapore longitudinal ageing study. Gerontology.

[45] Woodruff RC, Raskind IG, Harris DM, Gazmararian JA, Kramer M, Haardörfer R (2018). The dietary impact of introducing new retailers of fruits and vegetables into a community: results from a systematic review. Public Health Nutr.

[46] Barnes TL, Freedman DA, Bell BA, Colabianchi N, Liese AD (2016). Geographic measures of retail food outlets and perceived availability of healthy foods in neighbourhoods. Public Health Nutr.

[47] Rundle A, Neckerman KM, Freeman L, Lovasi GS, Purciel M, Quinn J (2009). Neighborhood food environment and walkability predict obesity in New York City. Environ Health Perspect.

[48] Bayat S, Widener MJ, Mihailidis A (2021). Bringing the “place” to life-space in gerontology research. Gerontology.

[49] Gesler WM, Albert DP. How spatial analysis can be used in medical geography. Spatial Analysis, GIS and Remote Sensing.CRC Press; 2000.

[50] Li J, Kim C (2018). Measuring individuals’ spatial access to healthy foods by incorporating mobility, time, and mode: activity space measures. Prof Geogr.

