# Supplementary material for: Availability of healthy foods, fruit and vegetable consumption, and cognition among urban older adults
Source: BMC Geriatr. 2023 May 17;23:302. doi: 10.1186/s12877-023-04003-z (PMC10189949; doi:10.1186/s12877-023-04003-z)
Supplement: Supplementary file 1 — Supplementary Material 1 [file 12877_2023_4003_MOESM1_ESM.docx]

# Supplementary Tables

Supplementary Table 1. Results from multilevel models: Separate analysis for objective and subjective availability of healthy foods.

1a) Symbol Match: Objective availability of healthy foods

| Outcome: Symbol Match | Estimate | SE | p |
| --- | --- | --- | --- |
| Intercept | 3.4404 | 0.1984 | <.0001 |
| Objective availability of healthy foods | -0.05589 | 0.04809 | 0.2451 |
| Linear session | -0.00789 | 0.001189 | <.0001 |
| Quadratic session | 0.000054 | 0.000013 | <.0001 |
| Linear time of day | 0.006156 | 0.000902 | <.0001 |
| Quadratic time of day | 0.001543 | 0.000157 | <.0001 |
| Age | 0.02845 | 0.01106 | 0.0101 |
| Female | -0.09397 | 0.106 | 0.3754 |
| nHB vs. nHW | 0.2725 | 0.1212 | 0.0245 |
| Other race vs. nHW | 0.2627 | 0.1408 | 0.0621 |
| Below HS vs. HS^a^ | 0.6581 | 0.2584 | 0.0109 |
| Associates/Bachelors vs. HS | -0.05334 | 0.1181 | 0.6517 |
| Graduate vs. HS | 0.01013 | 0.1359 | 0.9406 |
| Financial situation | -0.0268 | 0.02373 | 0.2589 |
| ADI | 0.01787 | 0.02131 | 0.4016 |
| Random effects |  |  |  |
| Var (Intercept) | 0.6807 | 0.06001 | <.0001 |
| Var (Session) | 0.000041 | 4.71E-06 | <.0001 |
| Var (Time) | 0.000093 | 0.000019 | <.0001 |
| Covar (Intercept, Session) | -0.00138 | 0.000396 | 0.0005 |
| Covar (Intercept, Time) | 0.000658 | 0.000804 | 0.4131 |
| Covar (Session, Time) | -2.50E-06 | 6.77E-06 | 0.7118 |
| Residual | 0.2809 | 0.00294 | <.0001 |

1b) Symbol Match: Subjective availability of healthy foods

| Outcome: Symbol Match | Estimate | SE | p |
| --- | --- | --- | --- |
| Intercept | 3.3433 | 0.1982 | <.0001 |
| Subjective availability of healthy foods | -0.1743 | 0.06012 | 0.0037 |
| Linear session | -0.00799 | 0.001208 | <.0001 |
| Quadratic session | 0.000055 | 0.000014 | <.0001 |
| Linear time of day | 0.006224 | 0.000912 | <.0001 |
| Quadratic time of day | 0.001547 | 0.000158 | <.0001 |
| Age | 0.03378 | 0.01117 | 0.0025 |
| Female | -0.1307 | 0.1071 | 0.2223 |
| nHB vs. nHW | 0.2568 | 0.1224 | 0.0359 |
| Other race vs. nHW | 0.2205 | 0.1373 | 0.1083 |
| Below HS vs. HS^a^ | 0.6811 | 0.249 | 0.0062 |
| Associates/Bachelors vs. HS | -0.03388 | 0.1183 | 0.7746 |
| Graduate vs. HS | 0.07789 | 0.1348 | 0.5634 |
| Financial situation | -0.00852 | 0.02507 | 0.734 |
| ADI | 0.01547 | 0.02072 | 0.4555 |
| Random effects |  |  |  |
| Var (Intercept) | 0.6633 | 0.05892 | <.0001 |
| Var (Session) | -0.00139 | 0.000395 | 0.0004 |
| Var (Time) | 0.000041 | 4.80E-06 | <.0001 |
| Covar (Intercept, Session) | 0.000697 | 0.000801 | 0.3845 |
| Covar (Intercept, Time) | -3.19E-06 | 6.89E-06 | 0.6431 |
| Covar (Session, Time) | 0.000093 | 0.000019 | <.0001 |
| Residual | 0.2832 | 0.002988 | <.0001 |

2a) Color Shape: Objective availability of healthy foods

| Outcome: Color Shape | Estimate | SE | p |
| --- | --- | --- | --- |
| Intercept | 0.5266 | 0.06201 | <.0001 |
| Objective availability of healthy foods | 0.02356 | 0.01566 | 0.1324 |
| Linear session | 0.004255 | 0.000542 | <.0001 |
| Quadratic session | -0.00003 | 6.07E-06 | <.0001 |
| Linear time of day | -0.00061 | 0.000363 | 0.0959 |
| Quadratic time of day | -0.00003 | 0.000062 | 0.5991 |
| Age | -0.00744 | 0.003738 | 0.0465 |
| Female | 0.008939 | 0.03517 | 0.7994 |
| nHB vs. nHW | -0.1271 | 0.04137 | 0.0021 |
| Other race vs. nHW | -0.04827 | 0.04813 | 0.3159 |
| Below HS vs. HS^a^ | -0.2136 | 0.08233 | 0.0095 |
| Associates/Bachelors vs. HS | 0.1058 | 0.04121 | 0.0103 |
| Graduate vs. HS | 0.1309 | 0.03922 | 0.0009 |
| Financial situation | 0.00248 | 0.00685 | 0.7173 |
| ADI | 0.01291 | 0.007208 | 0.0733 |
| Random effects |  |  |  |
| Var (Intercept) | 0.06372 | 0.005582 | <.0001 |
| Residual | 0.0844 | 0.000869 | <.0001 |

2b) Color Shape: Subjective availability of healthy foods

| Outcome: Color Shape | Estimate | SE | p |
| --- | --- | --- | --- |
| Intercept | 0.5545 | 0.06187 | <.0001 |
| Subjective availability of healthy foods | 0.0415 | 0.01687 | 0.0139 |
| Linear session | 0.004204 | 0.000549 | <.0001 |
| Quadratic session | -0.00003 | 6.16E-06 | <.0001 |
| Linear time of day | -0.00062 | 0.000369 | 0.094 |
| Quadratic time of day | -0.00003 | 0.000062 | 0.6197 |
| Age | -0.00871 | 0.003764 | 0.0207 |
| Female | 0.02014 | 0.03564 | 0.572 |
| nHB vs. nHW | -0.1245 | 0.04177 | 0.0029 |
| Other race vs. nHW | -0.03833 | 0.04826 | 0.427 |
| Below HS vs. HS^a^ | -0.2176 | 0.07948 | 0.0062 |
| Associates/Bachelors vs. HS | 0.1025 | 0.04147 | 0.0135 |
| Graduate vs. HS | 0.1148 | 0.03954 | 0.0037 |
| Financial situation | -0.00272 | 0.00683 | 0.6901 |
| ADI | 0.01237 | 0.007031 | 0.0786 |
| Random effects |  | Error |  |
| Var (Intercept) | 0.06343 | 0.0056 | <.0001 |
| Residual | 0.08506 | 0.000883 | <.0001 |

3a) Dot Memory: Objective availability of healthy foods

| Outcome: Dot Memory | Estimate | SE | p |
| --- | --- | --- | --- |
| Intercept | 2.3154 | 0.1737 | <.0001 |
| Objective availability of healthy foods | -0.08653 | 0.05191 | 0.0956 |
| Linear session | -0.0048 | 0.001527 | 0.0017 |
| Quadratic session | 8.60E-06 | 0.000017 | 0.623 |
| Linear time of day | 0.00407 | 0.001427 | 0.0043 |
| Quadratic time of day | 0.001003 | 0.000218 | <.0001 |
| Age | -0.00432 | 0.008863 | 0.6263 |
| Female | 0.4591 | 0.1014 | <.0001 |
| nHB vs. nHW | 0.2388 | 0.1062 | 0.0246 |
| Other race vs. nHW | 0.132 | 0.1395 | 0.3441 |
| Below HS vs. HS^a^ | 0.1911 | 0.1546 | 0.2164 |
| Associates/Bachelors vs. HS | -0.4734 | 0.109 | <.0001 |
| Graduate vs. HS | -0.6204 | 0.1215 | <.0001 |
| Financial situation | -0.01046 | 0.02054 | 0.6104 |
| ADI | 0.03134 | 0.02012 | 0.1193 |
| Random effects |  |  |  |
| Var (Intercept) | 0.5066 | 0.04492 | <.0001 |
| Residual | 1.0291 | 0.01058 | <.0001 |

3b) Dot Memory: Subjective availability of healthy foods

| Outcome: Dot Memory | Estimate | SE | p |
| --- | --- | --- | --- |
| Intercept | 2.2467 | 0.1795 | <.0001 |
| Subjective availability of healthy foods | -0.0769 | 0.05218 | 0.1405 |
| Linear session | -0.00469 | 0.001548 | 0.0025 |
| Quadratic session | 6.42E-06 | 0.000018 | 0.7177 |
| Linear time of day | 0.004275 | 0.001441 | 0.003 |
| Quadratic time of day | 0.001038 | 0.000218 | <.0001 |
| Age | -0.00067 | 0.008702 | 0.9385 |
| Female | 0.4241 | 0.1012 | <.0001 |
| nHB vs. nHW | 0.2331 | 0.1077 | 0.0304 |
| Other race vs. nHW | 0.1301 | 0.1329 | 0.3278 |
| Below HS vs. HS^a^ | 0.1905 | 0.1567 | 0.224 |
| Associates/Bachelors vs. HS | -0.4714 | 0.1099 | <.0001 |
| Graduate vs. HS | -0.5775 | 0.1237 | <.0001 |
| Financial situation | 0.003105 | 0.02189 | 0.8872 |
| ADI | 0.03494 | 0.02028 | 0.0849 |
| Random effects |  |  |  |
| Var (Intercept) | 0.5062 | 0.04523 | <.0001 |
| Residual | 1.034 | 0.01072 | <.0001 |
